# Obstetic Surgery

**Published:** 1895-03

**Authors:** 


### BOOK NOTICES.

OBSTETRIC SURGERY. By Egbert H. Grandin, M. D., Obstetric Surgeon to the New York Maternity Hospital, Gynæcologist to the French Hospital, etc.; and George W. Jarman, M. D., Obstetric Surgeon to the New York Maternity Hospital, Gynæcologist to the Cancer Hospital, etc.; with eighty-five (85) illustrations in the text and fifteen full-page photographic plates. Royal octavo, 220 pages. Extra cloth, $2.50, net. Philadelphia: The F. A. Davis Co., publishers, 1914 and 1916 Cherry Street.

As its name denotes, this book is a treatise upon the latest procedures in dystocia.

The author says in the preface: "The key-note of this volume is *election* in obstetric surgery. The results which are daily secured in general surgery through resort to timely operation are obtainable in obstetrics if the same principle be held in view."

Much stress is laid upon asepis and antisepsis. It is claimed that the liability to septicemia is largely reduced by care in asepsis, and that when it does occur it is entirely due to carelessness in the use of asepsis.

"Antisepsis," says the author, "is simply the means of certifying to asepis (cleanliness)."

"It is possible to secure asepsis without resorting to antisepsis, but in order to surround surgery with every possible safeguard chemical agents must be looked upon as absolutely essential. The point to be remembered in obstetric surgery is that too free indulgence in antisepsis may do harm even whilst it aims at good. The nature of many of the antiseptic agents on which we must needs rely is poisonous to the human body. Therefore, the corollary must be borne in mind that over-zealousness in matters of antisepsis may injure and kill, even as lack of asepsis may be followed by similar effects."

This quotation is specially selected for the readers of this journal because of its special significance to the homœpathist.

Elaborate and clear directions then follow for the securing of asepsis, and then follows the treatise upon dystocia in all its varied forms.

The determination of the pelvic diameters is given very carefully, with the aid of diagrams and photographic plates, and a careful analysis of the various deformities of the pelvis added. The production of artificial abortion and premature labor, together with the instruments needed, is described with considerable minuteness, and then follows a chapter on the forceps, well illustrated.

Chapter fourth treats of version, also elaborately illustrated. The critical operations of symphysiotomy, Cæsarean section, and embryotomy have chapters specially devoted to each one, and so, also, has the "Surgery of the Puerperium." This includes operations resulting from traumatism, such as laceration of the cervix, laceration of the pelvic floor, fistulæ, and rupture of the uterus, and affections depending upon septic infection, such as endometritis, metritis, pelvic abscess, peritonitis, and mastitis.

The final chapter treats of ectopic gestation, and the claim is made that by the method of treatment outlined ectopic gestation has been practically robbed of its terrors, and the absolute mortality rate of the past has been converted into the almost certain recovery rate of the present.

It will thus be seen that the author places his reliance for success in saving patients who have to pass through the ordeals of these various obstetric trials mainly upon the *prevention* of blood infection through absorption of germs from the atmosphere and the putrescent changes in the secretions of the womb and vagina. After that skill and judgment in the use of instruments and accurate knowledge of the mechanism of version are needed to insure a perfectly reliable operator, and the mission of this book is specifically *teaching* these requisites to insure a well-educated *accoucheur*.

